# Impact and mechanistic role of oral contraceptive pills on the number and epithelial type of ovarian cortical inclusion cysts; a clinicopathology and immunohistochemical study

**DOI:** 10.1186/s13000-016-0482-6

**Published:** 2016-03-22

**Authors:** Ali DastranjTabrizi, Parvin MostafaGharabaghi, Farzam SheikhzadehHesari, Liela Sadeghi, Sharareh Zamanvandi, Parvin Sarbakhsh, Morteza Ghojazadeh

**Affiliations:** Women’s Reproductive Health Research Center, Tabriz University of Medical Sciences-Iran, Tabriz, Iran; Department of Physiology, Faculty of Science, Tabriz University-Iran, Tabriz, Iran; Department of Statistic and Epidemiology, Faculty of Nutrition and Health, Tabriz University of Medical Sciences-Iran, Tabriz, Iran

**Keywords:** Oral contraceptive pills, Ovarian cortical inclusion cyst, Ovarian cancer

## Abstract

**Background:**

Ovarian epithelial cancers are among the most lethal women's cancers. There is no doubt about the preventive role of oral contraceptive pills (OCPs) in development of ovarian cancers. But, there are limited numbers of studies to address the effect of these agents on the number of cortical inclusion cysts (CICs), their epithelial type and suppression of the metaplastic phenomenon by these pills. The aim of this study was to clarify the role of these agents in the prevention of these cyst formation and tubal metaplasia and also examine the mesenchymal-epithelial transition theory in this context by immunohistochemical methods.

**Methods:**

The representative section(s) of ovarian cortex from a total number of 201 consecutive total abdominal hysterectomy with bilateral or unilateral salpingo-oophorectomy specimens were examined for mean number of CICs and their epithelial type between two groups of the patients. Group A included the patients who were on oral contraceptive pills for more than 5 years. All of the subjects with other contraceptive methods or a history of less than 5 years contraceptive pills usage were stratified in group B. Sections from 20 cases in which more than five inclusion cysts were found, were selected for IHC staining with calretinine and PAX8 as markers for mesothelium and mullerian epithelium respectively.

**Results:**

The mean age of the patients was 51.67 years with no significant differences between two groups. The mean number of cysts were 1.27 and 3.23 in group A and B respectively (*P* =0.0001). Similarly the mean number of CICs, lined by tubal epithelium, was significantly different between two groups (0.65 vs 2.65, *P* =0.0001). In IHC staining 123 out of 150 CICs (82 %) were PAX+ while only 7 CICs (4.8 %) showed positive reaction for calretinin irrespective of type of epithelium.

**Conclusion:**

Our findings showed that the use of OCP for more than five years in women, significantly prevents development of cortical inclusion cysts in the ovaries which lined by tubal (PAX8 positive) type epithelium. These findings may explain the alternative mechanism of oral contraceptive pills or long time use of progesterone in suppression of tubal type overgrowth and subsequently prevention of ovarian epithelial cancers.

## Background

Although ovarian cancer is considered as an uncommon malignancy, its mortality rate is the highest among gynecologic malignancies in developed countries and is second after cervical cancer in developing world [1–3]. Epithelial ovarian cancers (EOCs) are the fifth most common cancer in women who are living in UK and are responsible for more than 4400 death per year in this country [4, 5]. Age and genetics are the most well established risk factors for ovarian cancer [6, 7]. It most commonly occurs in peri or post-menopausal age group with the mean age of diagnoses occurring near 60 years. Hereditary tumors are often diagnosed approximately 10 years earlier [8, 9]. There is a clear association between family history for ovarian cancer and the risk of developing ovarian cancer in subsequent generations [8].

From historical perspective, it has been clarified that women who use oral contraceptive pills (OCP) are in lower risk for development of ovarian epithelial cancers. Based on many research results, women who have taken these agents are in one-third risk to develop ovarian cancer in comparison with non-users [10–13]. In the same way, the results of a more recently research showed that use of newly formulated pills with low estrogen and low progesterone level had the same risk reduction effect [14].

In regard to histogenesis of ovarian epithelial cancers particularly high grade serous carcinoma (the most lethal type of EOC) [15], one of the relevant theories indicates that epithelial inclusion cysts in the ovarian cortex may be affected by mutagenic and carcinogenic agents. These cysts may originate from ovarian mesothelium or implanted fimberial altered cells [16, 17]. The suppression of ovulation and decreased level of gonadotropin levels are considered the most important and relevant mechanisms for risk reducing effect of OCPs. But, the number of research studies to address the effect of these agents on the number of cortical inclusion cysts, their epithelial type and also suppression of *the metaplastic phenomenon by these pills is limited. As a known phenomenon in the endometrium OCPs have suppressive effects on glandular tubal metaplasia as well as other lesions that induced by unopposed estrogen effects. Similarly, use of high dose progesterone in treatment of endometrial hyperplasia and well differentiated endometriod carcinoma converts these abnormalities as well as tubal metaplasia to the normal phenotype. The primary aim of this cross-sectional clinicopathologic and imunohistochemical study was to address the preventive and suppressive mechanism(s) of OCPs on the development of tubal type epithelium (PAX8 positive cells) in the ovarian cortical cysts in OCP users and compare them with control group. The secondary aim was to characterize the immunohistochemical features of these cysts to clarifying this issue that, the ovarian cortex microenvironment induces tubal type (PAX8 positive) epithelium to these structures and confirm this hypothesis that regardless of the origin of these cysts, progesterone has the ability to suppress this molecular pathway and subsequently affects one of the pathways that probably lead to ovarian serous carcinogenesis. Finally, the last aim of this research was to link the suppressive role of progesterone agents on ovarian inclusion cyst lining and prevention of secretory cell outgrowth (SCOUT) in these cysts.*

## Methods

After Tabriz university of medical sciences ethical board approval, a cross sectional comparative study was conducted. A total number of 201 consecutive pathology specimens from patients who had been underwent to bilateral or unilateral salpigo-oophorectomy with or without hysterectomy at alzahra teaching hospital (Tabriz-Iran) from 2010 to 2012 were included. Based on the type of contraception and the duration of use, all of the patients stratified into the two main groups. Group A included the patients who were on OCP for more than 5 years (68 patients) and all of the subjects with other contraceptive methods or a history of less than 5 years use of OCP (133 patients) were included in the group B. The clinical data including age, marital status, parity, gravidity, age of menopause, past medical history, indication for surgery and particularly type of contraception and its duration gathered from patients medical records. The pathological findings extracted from patients pathology reports. The representative slides from each case retrieved from pathology file were used for examining the number of cortical inclusion cysts, type of their epithelium and presence or absence of tubal metaplasia in the ovarian surface epithelium. To unifying the number of cysts between the patients with BSO and USO, and also between the cases in which more than standard sections had been sampled, the mean number of the cysts in BSO group was taken into account. A total number of 20 cases in which more than five inclusion cysts were found in their representative sections, selected for IHC staining with calretinine and PAX8. We used these markers for identification of mesothelium and mullerian type epithelium, respectively. Data were analyzed by SPSS 21 soft ware and *P* value less than 0.05 considered for statistical significance.

### Immunohistochemistry

5 μm sections from paraffin-embedded tissues were mounted on microscope slides and underwent to heat-mediated antigen retrieval by using 750-800 W microwave oven. We used PH 9 and 6 for antigen retrieval solution in the retrieval process for PAX8 and calretinin respectively. Primary antibodies for detecting PAX8 (mouse monoclonal anti humanPAX8, clone BC12, Pleasanton CA.) and calretinin (mouse monoclonal, clone 2E7, Biogenic) were used and detected by manufacturing protocol.

## Results

The mean age of the 201 patients was 51.67 years (in range of 34 to76). The mean ages of the patients in case and control groups were 51.75 and 51.67 years with no significant difference between two groups. 3.26 % of cases presented with pelvic mass. Total abdominal hysterectomy was performed in 198 patients including 127 cases with bilateral salpingo-oophorectomy and 71 cases with unilateral salpingo-oophorectomy. In the remaining cases, the patients underwent for BSO or USO. The indications for surgery or tissue diagnosis after surgery in both groups of patients are shown in Table 1.

**Table 1:** Indication for surgery or tissue diagnosis in case and control groups

Indications for surgery or tissue diagnosis	Case (*n* = 68)	Control (133)
Leiomyoma	35	65
Ovarian mass	6	11
Ovarian cysts	6	20
Endometrial Carcinoma	3	13
AUB^a^	3	4
Adenomyosis	4	11
Endometrial Hyperplasia	0	3
Gestational Trophoblastic Disease	2	2
Atrophic or Unremarkable Endometrium	4	8
Cervical Intraepithelial Neoplasia	1	0

^a^Abnormal Uterine Bleeding

The mean number of cysts were 1.27 and 3.23 in group A and B, respectively (*P* =0.0001). The mean number of CIC with flat epithelium in both groups showing no significant difference. (0.63 *Vs* 0.94, *P* =0.11). However the mean number of CICs lined by tubal epithelium was significantly different between two groups (0.65 in group A vs. 2.65 in group B, *P* =0.0001). The mean number of the cysts with different epithelium between two groups is illustrated in Table 2. After adjustment of both groups for age and parity, we couldn't find any change in the results.

**Table 2:** Main number of CICs^a^ with different type of epithelium in both study groups

Group	Main number of CIC (%)	Main number of CIC with flat epithelium (%)	Main number of CIC with tubal epithelium (%)
A (*n* = 68)	1.27	0.63	0.65
B (*n* = 133)	3.23	0.94	2.65
*P* value	*P* =0.0001	*P* =0.11	*P* =0.0001

^a^Cortical inclusion cyst

**Fig. 1:**
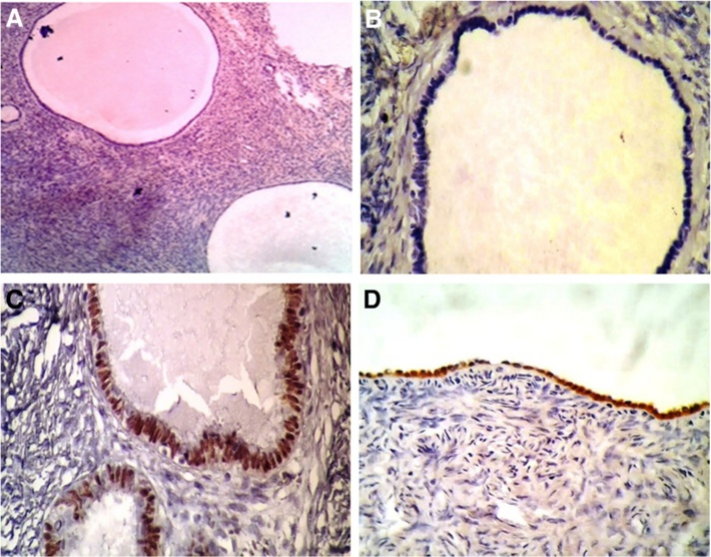
Negative staining for Calretinin in multiple CICs irrespective of their flat mesothelial type epithelium (**a**) Negative reaction for calretinin in a CIC with morphologically tubal type epithelium (**b**). PAX8 positivity in a CIC with tubal epithelium (**c**). Strong positive reaction for calretinin in ovarian surface epithelium (**d**)

**Fig. 2:**
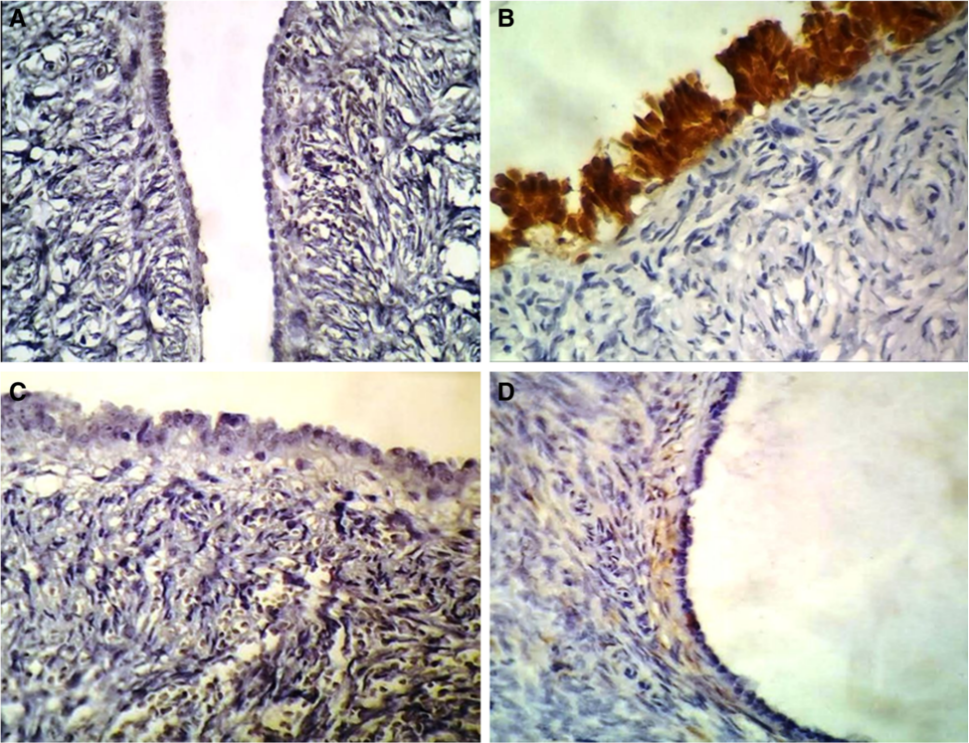
PAX8 negativity in a CIC with tubal and flat epithelium which has opened orifice (**a**). Calretinin positivity in the surface epithelium with tubal phenotype (**b**). PAX 8 negative reaction in surface epithelium with tubal phenotype (**c**). Negative reaction for calretinin in a CIC with closed lumen (**b**)

In regard to immunohistochemical findings a total number of 150 cortical inclusion cysts from 20 cases with highest number of CIC were stained with PAX8 and calretinin. 123 out of 150 CIC (82 %) were PAX+ while only 7 CIC (4.8 %) showed positive reaction for calretinin irrespective of tubal or flat epithelium (*p* = 0.001). In all of the 14 cases in which the surface epithelium was flat (mesothelial type), the reaction for PAX8 was negative while, in 12 cases, this type of epithelium was positive for calretinin (0 % vs 85 %). In contrast, in 11 cases, the metaplastic surface epithelium was negative for PAX8 (90 %), but, 20 % of the latter epithelium was calretinin positive (Table 3). Representative slides from both IHC staining are illustrated in Figs. 1 and 2.

**Table 3:** Immunohistochemical characteristics of ovarian CICs^a^ and surface epithelium in selected cases

Cases (20)	PAX8+ (%)	Calretinin + (%)	Missed cases in IHC sections	*P* value
CIC (*n* = 150)	123 (82 %)	7 (4.8 %)	20	*P* < 0.001
Surface Epithelium (Flat type, *n* = 14)	0 (0 %)	12 (85 %)	2	*P* < 0.001

^a^Cortical inclusion cysts

## Discussion

Epithelial ovarian cancers are the most common cancers of the ovary and among them; high grade serous carcinoma is the most lethal cancer of the female genital tract. The epithelial nature of these cancers mostly derived from the assumption that these tumors originate from ovarian surface epithelium (mesothelium) through malignant transformation of this epithelium or the lining of the CICs. Recent findings from numerous studies on histogenesis of ovarian high grade serous carcinoma strengthened this hypothesis that probably most of these highly malignant lesions arise from fallopian tube epithelium particularly from fimberial mucosa [18–21]. The term STIC (Serous Tubal Intraepithelial Carcinoma) introduced in the last decade for describing the tubal and fimberial mucosal change which occurs similar to other insitu carcinoma. In addition to finding of STIC in the most of fallopian tubes which removed for prophylactic purpose in patients who are carrier for BRCA1 or BRCA2, in 50 to 60% of sporadic cases of ovarian high grade serous carcinoma, this lesion has been detected by SEE-FIM protocol in the removed fallopian tubes also [22, 23].

Although the STIC hypothesis is very intriguing, however the relationship between use and particularly the role of OCP and prevention of ovarian carcinoma has not been addressed in this hypothesis. In the recent study published by Banet and kurman [17], they could not find any CIC with tubal type epithelium in the ovaries of premenarchal girls, whereas a trend in increasing number of ciliated type CICs with aging was seen. Presence of two types of CICs in their study and particularly absence of CIC with tubal epithelium before menarche lead them to this assumption that many of the ciliated type CIC develop during the time of ovulation. Although our findings are in line with their hypothesis, this assumption is considered an anecdotal mechanistic role of OCPs in prevention of ovarian epithelial cancers. As a new perspective, it is well known that the epithelial lining of the female genital tract is susceptible to tubal metaplasia particularly during unopposed estrogen exposure. For example tubal metaplasia is nearly a constant finding in the endometrial premalignant and malignant lesions and treatment of these lesions by progesterone agents usually suppresses the malignant epithelium and converts tubal epithelium to normal type. In this context, the significantly decreased number of the CICs lined by tubal type epithelium in the our case group support this new hypothesis that regardless of the site of origin, progesterone agents have the ability to suppress tubal metaplasia or overgrowth of the original tubal epithelium which has been implanted on the ovarian cortex and subsequently prevent the sequence of tubal metaplasia → secretary cells outgrowth → serous carcinoma.

Regard to our immunohistochemical results, we showed that almost all of the CICs epithelium in our study (both with tubal or flat epithelium) were calretinin negative and PAX8 positive whereas in 85 % of cases, in which the OSE was found, the surface epithelium was strongly positive for calretinin (Fig. 1, a - d). According to the Banet and Kurman’s hypothesis [18], this phenotypic change can be explained by this assumption that expansion of the CIC may cause flatting of the tubal type epithelium. In cases studied by Auersperg she found calretinin positive cells in the flat epithelium of the CICs and has indicated for transition zone between these two types of epithelium at least in some of these cysts [16]. The expression of PAX8 pathway in nearly all 150 stained CICs epithelium and suppression of this molecular signaling in OCP users should be explained in detail. In the control group who were not on long time OCPs, it can be explained in one hand by numerous episodes of ovulation that prone the ovarian cortex to formation of inclusion cysts or implantation of fimberial epithelial cells. In the other hand the suppressive effect of progesterone in the OCPs user group may affect the PAX8 pathway genetically and prevents tubal metaplasia or tubal type epithelium overgrowth. This phenomenon is a well recognized process in the endometrium and considered as a base for treatment of well differentiated endometrial carcinoma by high dose progesterone.

An alternative assumption which might explain these findings in all together is MET (Mesenchymal-epithelial transition) theory [24]. According to this hypothesis, the ovarian cortical microenvironment has a dramatic influence on the epithelium of CICs and induced epithelial markers expression on the migrated cells. Regardless of the origin of CIC epithelium from ovarian surface epithelium or fimberial implanted cells, it seems that the PAX8 pathway is expressed dominantly in the absence of progesterone, whereas the presence of relatively dominant level of progesterone may convert this pathway. As noted in the result section, 82 % of CICs, regardless of their lining phenotype, were positive for PAX8 while only 4.8 % of these CICs were calretinin positive (Fig. 1c). These findings were in line with previous research findings and confirm MET (Mesenchymal-epithelial transition) theory [24]. Interestingly our results also support the reverse phenomenon named epithelial –mesenchymal transition. Surprisingly in 20 % of IHC stained slides, the superficially located tubal type epithelium showed positive reaction for calretinin regardless of its phenotype. These findings are in line with the previous findings which indicate that presumably the high concentration of bioactive materials secreted by OSE in closed lumen of CICs induces epithelial markers acquisition regardless of their phenotypes [25–27] (Fig. 1c). In contrast when we encounter with surface epithelium invaginations which have opened orifice, the lining epithelium shows calretinin positivity and PAX8 negativity irrespective of their phenotype (Fig. 2a and b). In similar fashion, 90 % of cases in which the surface epithelium had acquired the tubal type phenotype, this epithelium were negative for PAX8 (Fig. 2c). Although the number of cases in relation to the latter findings were limited, these results confirm the effects of tissue microenvironment in acquisition or losing of cell markers regardless of their phenotype or origin.

## Conclusion

In summary our findings in the present study highlighted the progesterone role of OCPs in prevention of tubal metaplasia or tubal type epithelium overgrowth in the ovarian cortical inclusion cysts. We not only showed that the number of CICs lined by tubal epithelium has been decreased significantly in OCP users but also showed that probably progesterone of oral contraceptive pills suppresses the PAX8 pathway and prevents the sequence of tubal metaplasia → secretary cells outgrowth (SCOUT) → serous carcinoma. In addition, our IHC findings explain how the ovarian cortical microenvironment affects the nature of cortical cysts epithelium in the presence or absence of oral contraceptive pills.

## Abbreviations

BSO: bilateral salpingo-oophorectomy
CICs: cortical inclusion cysts
EMT: epithelial mesenchymal transition
EOCs: epithelial ovarian cancers
MET: mesenchymal epithelial transition
OCP: oral contraceptive pill
OSE: ovarian surface epithelium
SEE-FIM: sectioning and extensively examining of the fimberiated end
STIC: serous tubal intraepithelial carcinoma
TAH: total abdominal hysterectomy
USO: unilateral salpingo-oophorectomy
